# Author Correction: Extremely efficient aerogels of graphene oxide/graphene oxide nanoribbons/sodium alginate for uranium removal from wastewater solution

**DOI:** 10.1038/s41598-024-53952-x

**Published:** 2024-02-19

**Authors:** Ali A. Jabbar, Dhia H. Hussain, Kamal H. Latif, Salim Albukhaty, Adel Kareem Jasim, Ghassan M. Sulaiman, Mosleh M. Abomughaid

**Affiliations:** 1 College of Science/Chemistry Department, Mustansiriyah University, Baghdad, Iraq; 2 The Iraqi Authority for the Control of Radioactive Sources, Baghdad, Iraq; 3 Department of Chemistry, College of Science, University of Misan, Maysan, Iraq; 4 College of Medicine, University of Warith Al-Anbiyaa, Karbala, 56001 Iraq; 5 Division of Biotechnology, Department of Applied Sciences, University of Technology, Baghdad, 10066 Iraq; 6 Department of Medical Laboratory Sciences, College of Applied Medical Sciences, University of Bisha, 255, 67714 Bisha, Saudi Arabia

Correction to: *Scientific Reports* 10.1038/s41598-024-52043-1, published online 13 January 2024

The original version of this Article contained an error in Reference 10, which was incorrectly given as:

Imarah, A. A. *et al.* Graphene oxide-induced, reactive oxygen species-mediated mitochondrial dysfunctions and apoptosis: high-dose toxicity in normal cells. *Nanomedicine (London, England)* **18**(11), 875–887. 10.2217/nnm-2023-0129 (2023).

The correct reference is listed below:

Mohammed, H. A. *et al.* Solid lipid nanoparticles for targeted natural and synthetic drugs delivery in high-incidence cancers, and other diseases: Roles of preparation methods, lipid composition, transitional stability, and release profiles in nanocarriers’ development. *Nanotechnol. Rev.* **12**(1), 20220517 (2023).

In addition, in the Author Contributions section,

“A.A.J., D.H.H., K.H.L., performed the experiments and data analysis, S.A., A.K.J., G.M.S., M.M.A., prepared and characterized samples, M.S.J., A.M.A., S.A., G.M.S. A.G.A. writing-review and editing the manuscript.”

now reads:

“A.A.J., D.H.H., K.H.L., performed the experiments and data analysis, S.A., A.K.J., G.M.S., M.M.A., prepared and characterized samples, S.A., G.M.S. A.G.A. writing-review and editing the manuscript.”

The original Article has been corrected.

